# Phytochemical-based therapeutics from traditional eastern medicine: analgesic effects and ion channel modulation

**DOI:** 10.3389/fpain.2025.1537154

**Published:** 2025-01-31

**Authors:** Sung Eun Kim, Geehoon Chung, Sun Kwang Kim

**Affiliations:** Department of Physiology, College of Korean Medicine, Kyung Hee University, Seoul, Republic of Korea

**Keywords:** traditional medicine, phytochemicals, ion channels, analgesic, pain

## Abstract

Pain management remains a major challenge in the healthcare system. While synthetic analgesics are widely used for pain management, their effectiveness in managing chronic pain is often limited due to low efficacy or side effects. Thus, there is growing interest in exploring alternative pain relief methods, particularly using medicinal plants from traditional Eastern medicine and their phytochemicals. Previous studies have demonstrated the modulatory effects of various phytochemicals derived from herbal medicine on pain-related ion channels, such as voltage-gated sodium channels (Na_v_), calcium channels (Ca^2+^), and transient receptor potential (TRP) channels. Since these ion channels are integral to the transmission and modulation of pain signals, the ability of specific phytochemicals to activate or inhibit these channels presents a promising avenue for the development of novel analgesics. The goal of this review is to merge herbal insights with ion channel research to highlight the potential of natural compounds for safe and effective pain management. In this regard, we summarize the discovery and characterization of pain-relieving phytochemicals from herbal medicine, and we discuss their mechanisms of action and their potential to mimic or enhance the effects of conventional analgesics through ion channel modulation.

## 1 Introduction

Pain is a complex and multifaceted experience, serving as both a vital protective mechanism and, in many instances, as a persistent, debilitating condition. Defined as an unpleasant sensory and emotional experience associated with actual or potential tissue damage, pain is a multidimensional phenomenon that poses significant challenges in effective management (1, 2). While acute pain functions as a critical alert system for injury, chronic pain—often resulting from nerve damage or dysfunction in the nervous system—affects millions globally, severely disrupting physical, emotional, social, and psychological health. It remains one of the leading causes for seeking medical care. It imposes an immense societal and economic burden, reducing work productivity, increasing healthcare costs, and diminishing sufferers' quality of life (3). Despite the availability of numerous pain management options, current treatments for chronic pain are often insufficient, underscoring the urgent need for new therapeutic strategies that offer effective and sustainable relief (4). Pharmacological treatments, including opioids and nonsteroidal anti-inflammatory drugs (NSAIDs), remain the primary approaches to pain relief. However, these options are associated with significant drawbacks (5–7). Opioids, while highly effective for managing moderate to severe pain, carry high risks of addiction, tolerance, and physical dependence, often requiring escalating doses and increasing the likelihood of overdose, respiratory depression, and opioid use disorder (3, 8). On the other hand, NSAIDs are commonly used for inflammatory and mild to moderate pain but pose serious risks with long-term use, including gastrointestinal complications, cardiovascular events, and kidney damage—particularly in vulnerable populations such as the elderly or those with pre-existing conditions (9, 10). Gabapentin is widely used for pain and generally considered safe, but it has limitations, including the risk of respiratory depression, especially when combined with CNS depressants like opioids (11). These drawbacks emphasize the critical need for safer, more effective, and long-term pain management strategies that minimize harm while improving patients' quality of life (12).

Pain perception and its chronic manifestation are intricately linked to the function and dysregulation of ion channels, which play a pivotal role in transmitting pain signals within the nervous system. These channels, responsible for regulating the flow of ions such as sodium (Na^+^), potassium (K^+^), calcium (Ca^2+^), and chloride (Cl^−^) across cell membranes, are essential for maintaining neuronal excitability and synaptic transmission (13, 14). Dysregulation or sustained activation of these channels, frequently observed in chronic pain states, results in heightened neuronal excitability and abnormal pain signaling. Consequently, targeting ion channels has emerged as a promising therapeutic approach for pain management, with the potential to modulate pain pathways more precisely and with fewer side effects compared to conventional pharmacological treatments (15). Understanding the dynamics of ion channels is, therefore, critical for developing innovative, effective, and safer analgesics for chronic pain relief.

There has been growing interest in alternative pain management therapies, particularly those derived from natural sources such as traditional Eastern medicinal herbs (16). Eastern medicinal herbs have been used for centuries to treat pain and inflammation, offering a plant-based alternative that is gaining recognition for its potential efficacy and safety (17–19). These herbs contain bioactive compounds that can influence pain pathways, making them especially promising for managing chronic conditions (20–22). Recent studies highlight the role of ion channels in pain mechanisms and reveal the molecular pathways mediating the analgesic effects of herbal extracts. These compounds can exert their pain-relieving effects by targeting a single ion channel or simultaneously acting on multiple ion channels, enhancing their analgesic potential.

This review seeks to provide a comprehensive analysis of the efficacy of medicinal herbs in pain management by examining a wide range of scientific studies. It will explore the key ion channels involved in pain transmission and their interactions with specific medicinal herbs that contribute to analgesic effects. Additionally, this review will systematically address how these interactions influence various types of pain. By elucidating the relationship between medicinal herbs and pain-related ion channels, this study aims to identify potential alternatives to conventional pain treatments and pave the way for alternative therapeutic strategies.

## 2 Ion channels in pain pathways

Many ion channels including voltage-gated sodium channels (Na_v_), voltage-gated calcium channels (Ca_v_), ATP-sensitive potassium channels (K_ATP_), voltage-gated potassium channels (K_v_), transient receptor potential (TRP) channels, purinergic receptors such as P2X_3_, P2X_4_ and P2X_7_, acid-sensing ion channels (ASICs), play significant roles in pain signaling. Herbal medicines contain multiple bioactive compounds that can simultaneously interact with these ion channels, not only targeting individual channels but also providing a multi-faceted approach to pain management. By modulating multiple ion channels involved in pain transmission, these natural compounds offer a broader and more effective approach to analgesia (23). Understanding these channels paves the way for developing more targeted pain therapies (24–26).

### 2.1 Voltage-gated sodium channels (Na_v_)

One of the most prominent ion channels involved in pain signaling is the voltage-gated sodium channel (Na_v_) family, which facilitates the rapid influx of Na^+^ during the depolarization phase of action potential (AP) (27). Na_v_ channels are divided into tetrodotoxin-sensitive (TTX-S) channels such as Na_v_1.1, Na_v_1.2, Na_v_1.3, Na_v_1.6, and Na_v_1.7, and tetrodotoxin-resistant (TTX-R) channels such as Na_v_1.8 and Na_v_1.9. While Na_v_1.1, Na_v_1.2 are primarily expressed in the central nervous system (CNS) and involved in central sensitization, Na_v_1.7 is predominantly expressed in the peripheral nervous system (PNS) and is critical for pain transmission (28). Na_v_1.6 channels are expressed in both the central and peripheral nervous systems, functioning as the major Na_v_ isoform at the nodes of Ranvier while also being present in unmyelinated fibers and at the nerve terminals of certain sensory neuron subsets (29). TTX-R channels, including Na_v_1.8 and Na_v_1.9, are predominantly found in PNS, mediating inflammatory and neuropathic pain. Na_v_1.8 propagates AP under inflammatory conditions, while Na_v_1.9 modulates chronic pain excitability. Na_v_1.5, though mainly cardiac, may have a peripheral role requiring further study. Alterations in their expression or function contribute to chronic pain sensitization, with Na_v_ 1.7, Na_v_ 1.8, and Na_v_ 1.9 gene variants linked to pain disorders, making them promising targets for pain management (27, 30, 31). Recent studies aim to develop selective Na_v_ channel inhibitors for effective pain relief with fewer systemic side effects, highlighting their importance in pain signaling and potential for targeted pain management strategies (32).

### 2.2 Voltage-gated calcium channels (Ca_v_)

Voltage-gated calcium channels (Ca_v_) play a pivotal role in the process of pain perception by regulating the release of neurotransmitters at synaptic terminals. These channels facilitate the entry of calcium ions into neurons in response to membrane depolarization, which is essential for the release of neurotransmitters such as glutamate, a key excitatory neurotransmitter in the pain pathway (33). L-type calcium channels, as high-voltage-activated channels, contribute to central sensitization and chronic pain by enhancing synaptic transmission and neuronal excitability. While not directly involved in acute pain, their dysregulation can amplify pain signals in neuropathic and inflammatory pain (34, 35). T-type calcium channels, especially Ca_v_3.2, are low-voltage-activated and play a critical role in nociceptive transmission. Highly expressed in peripheral sensory neurons, they facilitate neuronal hyperexcitability in acute and chronic pain states, making them promising therapeutic targets for pain relief (35–38). Both L-type and T-type channels are crucial in pain signaling, with L-type channels contributing to chronic pain and T-type channels driving nociception and sensitization, offering distinct yet complementary roles in pain modulation (34, 39).

### 2.3 ATP-sensitive potassium channels (K_ATP_) & voltage-gated potassium channels (K_v_)

K^+^ channels are integral in maintaining the resting membrane potential and controlling the excitability of neurons, including those involved in pain transmission. They include various subtypes, such as ATP-sensitive potassium channels (K_ATP_) and voltage-gated potassium channels (K_v_) (40). Activation of these channels generally results in an efflux of K^+^ from neurons, leading to hyperpolarization of the membrane, which makes it less likely for the neuron to reach the threshold required for AP generation. K_ATP_ channels, found in both peripheral and central neurons, are modulated by metabolic states and have been shown to play a role in the pain associated with ischemic conditions (41). K_v_ channels, such as K_v_7 (KCNQ), are also critical in stabilizing neuronal membrane potential. Modulation of K_v_7 channels has emerged as a promising approach for pain relief, as selective activators of these channels can reduce hyperexcitability in nociceptive neurons (42). Their activation generally reduces neuronal excitability, making them a potential therapeutic target for conditions characterized by hyperexcitability, such as chronic pain. By modulating potassium channel activity, it is possible to reduce the firing of nociceptive neurons, thereby decreasing pain perception.

### 2.4 Transient receptor potential (TRP) channels

TRP channels form a diverse family of ion channels that are sensitive to various physical and chemical stimuli, making them key players in pain detection and transmission. Among the TRP family, transient receptor potential vanilloid 1 (TRPV1), transient receptor potential ankyrin 1 (TRPA1), and transient receptor potential melastatin 8 (TRPM8) are particularly relevant to pain research, as they mediate responses to noxious heat, cold, and chemical irritants. TRPV1 is activated by noxious heat (above 43°C), capsaicin, and acidic conditions, which are often associated with tissue injury and inflammation (23). Interestingly, prolonged TRPV1 activation leads to desensitization, reducing pain signaling, which is exploited in treatments like capsaicin cream for neuropathic pain. However, excessive activation can paradoxically cause hyperalgesia or inflammation, highlighting its dual role in pain modulation (43). TRPA1 is typically co-localized with TRPV1 and is activated by irritants, inflammatory mediators, cold, and mechanical stimuli, serving as a molecular integrator for pain and neurogenic inflammation (43, 44). On the other hands, TRPM8 utilizes a distinct analgesic mechanism, being activated by cool temperatures and menthol, which contribute to the sensation of cooling and cold-induced pain relief. Modulating TRPM8 activity has been investigated as a therapeutic approach, particularly for alleviating conditions associated with burning pain or heat hyperalgesia (45–47). Together, these channels represent critical targets for the development of novel analgesic therapies, offering distinct and complementary mechanisms for addressing various types of pain.

### 2.5 Acid-sensing ion channels (ASICs)

ASICs are proton-gated ion channels that are activated by decreases in extracellular pH, which often occur in response to tissue damage or inflammation. These channels are highly expressed in peripheral sensory neurons, where they play a role in detecting pain associated with acidosis, such as that seen in ischemic or inflammatory conditions. When tissue injury leads to a drop in pH, ASICs open, allowing Na^+^ to enter the neuron, which contributes to the sensation of pain (48–50). The contribution of ASICs to hyperalgesia has made them a target of interest for pain research, as blocking these channels can reduce pain in conditions where tissue acidosis is a major factor. For example, in animal models of inflammatory pain, pharmacological inhibition of ASICs has been shown to alleviate hyperalgesia, indicating their potential as therapeutic targets for treating chronic pain (51).

### 2.6 Purinergic receptors P2X_3_, P2X_4_ and P2X_7_

ATP released from damaged or inflamed tissues activates P2X receptors on primary afferent neurons, leading to depolarization and the initiation of pain signals. These ATP-dependent ligand-gated cation channels are upregulated following nerve injury. P2X_3_ receptors, expressed in small-diameter sensory neurons, contribute to acute nociception, while P2X_2/3_ receptors modulate prolonged sensitivity associated with nerve injury or inflammation (52). The P2X_7_ receptor, expressed in both the nervous and immune systems, plays a critical role in pain development by mediating the release of inflammatory cytokines through ATP activation and intracellular signaling. Its involvement in inflammatory responses and pain modulation has been widely recognized and validated (53). Inhibiting P2X_4_ receptor function or expression, as well as targeting its regulatory molecules, has shown promise in suppressing neuropathic pain, making P2X_4_ receptors a critical therapeutic target (54, 55). P2X_3_ receptors contribute to acute nociception, while P2X_2/3_ receptors modulate prolonged sensitivity linked to nerve injury or inflammation. In neuropathic pain, P2X_4_ on microglia maintains nociceptive sensitivity via neuronal-glial interactions, and antagonists targeting these receptors have shown efficacy in reducing pain (56). Further research into the structure, function, and pharmacological inhibitors of P2X_4_ receptors could advance targeted therapies for chronic pain, addressing a significant challenge in pain medicine.

## 3 Phytochemicals and ion channels: insights from herbal medicine for pain management

We classify plants into four main types based on the parts used—roots, stems, leaves, and fruits. This categorization highlights the diverse compounds found in each plant part and their specific interactions with ion channels, which contribute to their analgesic effects (Tables 1–3).

**Table 1:** The effects of roots on pain models and ion channels.

Herbs & active compounds	Pain model (type of pain induced) & *in vitro*	Dosage (mg/kg) & route	Targeted ion channel& receptor	Ref
*Aconiti Brachypodi*	Hot plate test, writhing test, formalin test in female mice *In vitro* rat DRG neurons	1–20 mg kg, *i.g.*	TTX-S Na_v_ ↓	(57, 58)
*Aconitum* (Bulleyaconitine A)	Paclitaxel-induced neuropathic pain	0.1, 0.4, 0.8 mg/kg, *i.g.*	Na_v_1.7, Na_v_1.8 ↓	(59)
*Allium macrostemon*	Acetic-induced &formalin-induced model, hot plate test, *In vitro* HEK-293T cells, mouse DRG neurons	50, 100 mg/kg, *i.*p. *in vitro* 50 mg/L	Na_v_1.7 ↓	(60)
*Angelica dahurica* (Osthole)	CFA-induced pain, heat and capsaicin-induced pain model *In vitro* mouse DRG neurons	100 mg/kg, 600 mg/kg, *p.o.*	TRPV1 ↓	(61)
*Angelica dahurica* (Furanocoumarins)	Formalin-induced pain, capsaicin-induced pain in rats *In vitro* DRG neurons form TRPV1 ^−/−^ mice	3.45 µM; 30 µl, *s.c*. 400 µM; 40 µl (directly into the right eye) *in vitro* 50 µM	TRPV1 ↓	(62)
*Angelica sinensis* (Ferulate)	CCI in rats	50 mg, 100 mg/kg, *i.*p*.*	P2X_3_ ↓	(63)
*Angelicae pubescentis* (Coumarin)	SNI in rats	20 mg/kg, *i.g.*	TRPV1 ↓	(64)
*Angelicae pubescentis* (Columbianadin, Osthole)	*In vitro* mouse DRG neurons	*in vitro* 100 µM	T-,L- type Ca^2+^ ↓	(65)
*Asarum sieboldii* (Eugenol)	*In vitro* transfected in CHO cells	*in vitro* 625 µM	Na_v_1.7 ↓	(66)
*Bupleurum chinense* (Saikosaponins)	CCI in rats, formalin-induced pain in mice	2.5, 5.0, 10.0 mg/kg, *i.*p.	Na_v_1.7 ↓	(67)
*Cinnamomum* (Coumarin, Cinnamaldehyde)	*In vitro* mouse DRG neurons	*in vitro* 15, 30, 60 µM	TRPV1↑, TRPM8↓ TRPA1↑	(68)
*Corydalis yanhusuo* extracts	Formalin-induced pain in mice *In vitro* transfected in CHO cells	3.6, 6, 10, 20, 40 mg/kg, *i.*p.	Na_v_1.7↓	(69)
*Curcuma* (Curcumin)	Formalin-induced flinching behavior, vincristine-induced model *In vitro* rat DRG neurons	3.1–100 mg/kg, *p.o.*	K_ATP_ ↑ TRPA1↓ P2X_3_↓	(70, 71)
*Dioscorea bulbifera* (Diosbulbin)	CFA induced model in mice, PSNL models	250–500 mg/kg, *p.o.*	NO-cGMP-ATP sensitive K^+^ ↑	(72)
*Ginseng* (Ginsenosides, Gintonin)	*In vitro* rat DRG neurons Xenopus oocytes expressing hK_v_1.2	*in vitro* 100 µg/ml	L-, N- type Ca^2+^ ↓ K_v_1.2↓	(73, 74)
*Glycyrrhiza uralensis* Fisch (Licorice)	Formalin-induced pain model in mice *In vitro* DRG neurons transfected on HEK293T cells	25 mg/kg, *s.c*. *in vitro* 30 µM	Na_v_1.7 ↓	(75)
*Ligusticum Chuanxiong Hort* (Ligustrazine)	*In vitro* mouse DRG neurons in chronic venous disease model	*in vitro* 600 µM	TRPA1 ↓	(76)
*Scutellaria baicalensis* (Baicalein, Wogonin)	*In vitro* transfected rat TREK-2 in COS-7 cells	*in vitro* 100 µM	TREK-2 ↑	(77)
*Sinomenium acutum* (Sinomenine)	Formalin-induced pain model *in vitro* mouse DRG neurons	50 mg/kg, *i.*p*.*	Na_v_ ↓	(78)
*Sophorae radix* (Sophora flavanone G)	*In vitro* Ca_v_3.1 or Ca_v_3.2 stably expressed in HEK 293 cells	*in vitro 3 *µM	Ca_v_3.2 T-type Ca^2+^ ↓	(79)
*Pueraria montana* (Puerarin)	Von Frey test, Hargreaves’ test in CCI rats *In vitro* DRG neurons from paclitaxel-induced pain rats	*in vitro* 1 µM, 10 µM	Na_v_1.8, P2X_3_ ↓	(80, 81)
*Paeonia lactiflora* (Paenoiflorin)	*In vitro* DRG neurons from CFA-induced pain in mice NG108-15 cells	30 mg/kg, *i.*p. *in vitro* 30 µM	TRPV1, L-type Ca^2+^ ↓	(82, 83)

**Table 2:** The effects of stems & leaves on pain models and ion channels.

Herbs & active compounds	Pain model (type of pain induced) & *in vitro*	Dosage (mg/kg) & route	Targeted ion channel& receptor	References
*Artemisia annua* (Artemisinin)	CCI rat models *In vitro* transfected in HEK293 cells & DRG neuron in CCI models	5 mg/kg, *i.*p. *in vitro* 0.1–10 µM	P2X_4_ ↓	(84)
*Boswellia carterii and Commiphora myrrha* (Frankincense and myrrh)	CCI in mice *In vitro* DRG neuron from CCI models in mice	1.5 g/kg, *i.g.*	TRPV1↓	(85, 86)
*Camellia sinensis* (Epigallocatechin gallate)	Acetic acid-induced pain model in mice	100 µM/L, *i.pl.*	ASIC3↓	(87)
*Citrus reticulata* extracts	*In vitro* hTRPV1 overexpressed HaCaT-TRPV1-overexpressed cells HEK293 cells transfected hTRPA1, rTRPV1 DRG neuron in TRPV1/TRPA1^(−/−)^ rats		TRPV1, TRPM3, TRPA1 ↓ Na_v_1.7, Na_v_1.8 ↓	(88, 89)
*Ephedra sinica* (Ephedrine)	Capsaicin-induced pain in mice	700 mg/kg, *p.o.*	TRPV1↓	(90)
*Hericium erinaceus* extracts	SNL induced pain in mice SH-SY5Y cells	100 mg/kg, *p.o.*	P2X_4_, P2X_7_ ↓	(91)
*Magnolia officinalis* (Magnolol, Honokiol)	Tail-flick, hot-plate, formalin tests in mice *In vitro* NG108-15 cells	5, 10 mg/kg, *i.*p. *in vitro* 30 µM	Na_v_, K_v_ ↓	(92, 93)
*Mentha arvensis* (Menthol)	Von Frey test in mice *In vitro* rat DRG neurons F11 cells	20 µl of 1 mM stock, s.c. *in vitro* 125 µM–500 µM	Na_v_1.8, Na_v_1.9↓ TTX-S Na_v_ ↓	(94)

**Table 3:** The effects of fruits & flowers on pain models and ion channels.

Herbs & active compounds	Pain model (type of pain induced) & *in vitro*	Dosage (mg/kg) & route	Targeted ion channel & receptor	Ref
*Crataegus pinnatifida* (Vitexin)	Acetic acid-induced writhing, formalin-induced, CFA- induced model in mice	1, 3, 10 mg/kg, *i.*p*.*	TRPV1↓	(95)
*Lycium barbarum*	DSS-induced ulcerative colitis in rats	100 mg/kg via gavage	TRPV1, TRPA1↓	(96)
*Inula Britannica* (Essential oil, Patuletin)	Tail-flick, writhing, formalin-induced, and glutamate-induced tests in mice	25, 50, 100 mg/kg*, i.*p. 30 mg/kg, *i.*p.	NO-cyclic GMP-protein kinase G/ATP-sensitive K^+^↑	(12, 122)
*Garcinia mangostana* (α-Mangostin)	*In vitro* DRG neuron in mice, ND7/23 cells, Overexpressed K2P channels in HEK 293 cells	*in vitro* 0.3–3 mM	TRPV1, TTX-S Na_v_ ↓ TREK- 1/2, TRAAK↑	(97)
*Tetradium daniellii* (Pellitorine)	*In vitro* transfected HaCa-T cells	*in vitro* 3.75 mM	TRPV1 ↓	(98)
*Rhododendron molle* G. Don (Rhodojaponin III)	Hot plate, tail-immersion, acetic acid writhing, and formalin tests in mice and rats *In vitro* hNa_v_1.5-CHL, hNa_v_ 1.7-HEK293, and hNa_v_ 1.8-HEK293	0.025–0.30 mg/kg, *i.g*. *in vitro* 22.22–200 µM	Na_v_1.7, Na_v_1.8, Na_v_1.5↓	(99)

### 3.1 Roots

#### 3.1.1 *Aconitum* (neoline, bulleyaconitine A)

*Aconitum* which has traditionally been used for pain relief, including rheumatism and neuralgia, due to their potent anti-inflammatory and analgesic effects, making them valuable for managing chronic and inflammatory pain conditions (100, 101). Aconitum species have been used as medicinal herbs, and their various components have been reported to demonstrate analgesic effects. *Aconiti Brachypodi* Radix*,* derived from the dried roots of *Aconitum brachypodum* Diels (Family Ranunculaceae), is particulary renowned for its anti-rheumatic and analgesic properties. Extracts from *Aconiti Brachypodi* Radix show analgesic effects *in vivo*, demonstrated through hot-plate, writhing, and formalin tests. *In vitro*, it suppresses TTX-S sodium currents in rat DRG neurons. These results suggest that the analgesic effect may be linked to the modulation of TTX-S sodium currents in sensory neurons (57, 58). Neoline, an active ingredient, effectively alleviates oxaliplatin-induced neuropathic pain, including mechanical and cold hyperalgesia, by improving neurite elongation in DRG neurons. It modulates pain-related ion channels and relieves pain without causing sedation or motor impairment (102, 103). Bulleyaconitine A, an active ingredient of *Aconitum bulleyanum*, is known for its long-lasting analgesic effects by modulating voltage-gated sodium channels, particularly Na_v_1.7 and Na_v_1.8, and blocking TTX-S sodium channels in DRG neurons through protein kinase C (PKC) inhibition, effectively reducing neuropathic and chronic pain (59). Its action is more potent in neuropathic conditions due to upregulated sodium channels and PKC. Also, as well as Neoline, Bulleyaconitine A attenuates paclitaxel-induced neuropathic pain (104). Additionally, it modulates spinal microglia, enhances morphine's analgesic effects without affecting acute pain, and induces antinociception in rats and mice through alkaloids from *Aconitum* (105, 106).

#### 3.1.2 Allium macrostemon

*Allium macrostemon* is traditionally used for thoracic pain and heart-related conditions, known for antioxidant and vasodilatory benefits, though its analgesic effects are unstudied. Recent findings, using HEK293T cells and formalin-induced, acetic acid-induced, and thermal pain models, support its potential for pain relief and development as a Na_v_1.7-targeted analgesic (60).

#### 3.1.3 *Angelica dahurica* (imperatorin, osthole)

*Angelica dahurica*, a traditional herb from the Apiaceae family, is commonly used for treating headaches, toothaches, and skin issues. *Angelica dahurica* extract effectively reduces mechanical and thermal hypersensitivity in CFA-induced inflammatory pain in mice. Osthole, an extract from *Angelica dahurica*, directly inhibits TRPV1 activity in DRG neurons and reduces noxious heat- and capsaicin-induced pain in mice, with calcium imaging studies further demonstrating its potential as a promising analgesic for chronic inflammatory pain (61). Furanocoumarin imperatorin is the main active component that inhibits formalin- and capsaicin-induced pain in rats by acting as a weak agonist of the TRPV1 channel, likely binding near the capsaicin site and delaying desensitization recovery. These findings highlight imperatorin's potential as a lead compound for developing TRPV1-targeted pain treatments (62).

#### 3.1.4 *Angelica sinensis* (ferulate)

The roots of *Angelica sinensis* are famous for their use in pain relief, particularly for gynecological conditions and inflammation (107). Sodium ferulate, a major active compound known for its antioxidant and anti-inflammatory properties, has been widely used in the treatment of cardiovascular and cerebrovascular diseases. Recent studies have investigated its effects on hyperalgesia in a chronic constriction injury (CCI) rat model. It significantly increased the mechanical withdrawal threshold and thermal withdrawal latency, indicating reduced pain sensitivity. Moreover, Sodium ferulate's effect on hyperalgesia was mediated through the modulation of the P2X_3_ receptor in primary sensory afferents. While CCI elevated P2X_3_ receptor expression in DRG neurons, Sodium ferulate effectively reduced this upregulation, suggesting its potential for alleviating thermal and mechanical hyperalgesia during chronic neuropathic pain (63).

#### 3.1.5 *Angelicae pubescentis* (columbianadin, osthole)

The roots of *Angelicae pubescentis* have been widely used in traditional medicine to relieve pain associated with arthritis, rheumatism, and muscular discomfort (108). The extracts of *Angelicae pubescentis* reduce behaviors of acute pain, formalin-induced inflammatory pain, and neuropathic pain in a spared nerve injury (SNI) model and coumarins in the extract are the active anti-nociceptive components (109, 110). The coumarins, key active components derived from roots, are renowned for their anti-inflammatory and analgesic properties. They have been shown to significantly alleviate neuropathic pain and suppress the development of mechanical hypersensitivity induced by SNI. The anti-nociceptive effects of coumarins are linked to their regulation of pro-inflammatory cytokines, including TNF-α, IL-1β, and IL-6, as well as their modulation of TRPV1 and pERK pathways in the peripheral nervous system (64). Among the components of coumarins with demonstrated analgesic effects, columbianadin has been shown to inhibit acute and inflammatory pain behaviors. It also suppresses mechanical and cold hypersensitivity induced by oxaliplatin (109). Additionally, osthole, another coumarin compound, may reduce neuropathic pain behaviors by inhibiting T- and L-type calcium channels in nociceptive DRG neurons in mice (65).

#### 3.1.6 *Asarum sieboldii* (eugenol)

The roots of *Asarum sieboldii* are used for local anesthetics treat to toothache, headache, and inflammatory diseases. Methyl eugenol (4-allyl-1,2-dimethoxybenzene), a major component extracted from *Asarum sieboldii* exhibits antinociceptive effects in mice, as shown in the formalin-induced pain test, and reduces NMDA receptor-mediated hyperalgesia through GABA receptors (111). Na_v_1.7 channels which are TTX-S channels were inhibited by methyl eugenol, demostrated by whole-cell patch clamp experiments in CHO cells (66).

#### 3.1.7 *Bupleurum chinense* (saikosaponins)

*Bupleurum chinense* are rich in compounds like saponins, volatile oils, and flavonoids. Its main active ingredient, saikosaponin*,* has shown various pharmacological effects, including anti-inflammatory, analgesic, and hepatoprotective actions. Saikosaponin, reduces neuropathic pain in CCI rats via p38 MAPK and NF-*κ*B pathways, while saikosaponin A and D alleviate inflammatory pain in carrageenan-induced rats by inhibiting the NF-*κ*B pathway, producing pro-inflammatory mediators (112, 113). Saikosaponin inhibited Na_v_1.7, reducing thermal pain and decreased pain responses in phase 2 of the formalin-induced pain model *in vivo* (67).

#### 3.1.8 *Cinnamomum cassia* (coumarin, cinnamic acid, cinnamaldehyde)

Cinnamomi Cortex (bark of *Cinnamomum cassia* Presl) effectively alleviates oxaliplatin-induced cold allodynia in rats. It reduces cold allodynia and suppresses spinal glial and pro-inflammatory cytokine activation, with coumarin contributing to these effects. Cinnamic acid, a major component of *Cinnamomum cassia*, is particularly effective in reducing cold and mechanical hypersensitivity by inhibiting spinal pain transmission (114, 115). Cinnamomi Cortex has warming properties and influences pain-related pathways by modulating TRP channels. Key compounds like cinnamaldehyde increase TRPV1 and decrease TRPM8 expression in DRG neurons (116), and activate cold-sensitive TRPA1 channels, which raises cytoplasmic Ca^2+^ levels, enhancing cellular function and warmth effects (68, 117).

#### 3.1.9 Cordyalis yanhusuo

*Corydalis yanhusuo* extracts and its active component, tetrahydropalmatine, alleviate neuropathic pain. Tetrahydropalmatine has antinociceptive effects in acute and chronic pain models, specifically inhibiting the second phase of formalin-induced pain when administered intraperitoneally (118). *Corydalis yanhusuo* contains active alkaloid components that target Na^+^ ion channels, particularly Na_v_1.7 and Na_v_1.5, which may contribute to its analgesic and anti-arrhythmic effects (69). Molecular docking and patch clamp studies revealed that dihydrosanguinarine and dihydrochelerythrine, other active compounds, inhibit peak currents and modulate activation phases of these channels, supporting potential therapeutic applications in pain (119).

#### 3.1.10 *Curcuma* (curcumin)

The rhizomes of *Curcuma longa* (turmeric) are widely used for their potent anti-inflammatory, anti-cancer, antioxidant, and analgesic properties. Curcumin [1,7-bis(4-hydroxy-3-methoxyphenyl)-1,6-heptadiene-3,5-dione], the primary active compound, not only activates K_ATP_ channels, contributing to its antinociceptive effects but also specifically modulates the TRPA1 by activating and desensitizing it. This dual action on K_ATP_ and TRPA1 channels, both critical in pain perception, underscores curcumin's analgesic potential in managing various chronic pain conditions (70). Curcumin significantly attenuated vincristine-induced neuropathy, likely due to its combined antinociceptive, calcium-inhibitory, and antioxidant effects (120). Also, some studies have shown that curcumin effectively reduces neuroinflammation-driven chronic pain by modulating microglia and astrocytes and suppressing pathways like MAPK, NF-κB, and JAK-STAT. This modulation decreases pro-inflammatory mediators and enhances anti-inflammatory responses, making curcumin effective in treating neuropathic and inflammatory pain (121). Furthermore, nanoparticle-encapsulated curcumin (nano curcumin) has shown efficacy in reducing mechanical and thermal hyperalgesia in HIV-gp120-induced pain models by inhibiting P2X_3_ receptor activation and decreasing ERK1/2 phosphorylation in rat DRG neurons. This suggests that nano curcumin may be an effective strategy for mitigating neuropathic pain through P2X_3_-mediated pathways (71).

#### 3.1.11 *Dioscorea bulbifera* (diosbulbin)

The methanolic extract of *Dioscorea bulbifera* var sativa showed significant antinociceptive effects in both inflammatory and neuropathic pain models. It effectively reduced persistent pain induced by CFA and neuropathic pain through partial sciatic nerve ligation (PSNL). The extract also inhibited acute LPS-induced pain, although it had limited effects on thermal hyperalgesia and capsaicin-induced nociception. The antinociceptive effects in the PGE2-induced hyperalgesia model were reversed by L-NAME and glibenclamide, indicating a mechanism involving activation of the NO–cGMP–ATP-sensitive potassium channels pathway. *Dioscorea bulbifera* may offer therapeutic potential for managing both inflammatory and neuropathic pain (72, 122).

#### 3.1.12 *Ginseng* (ginsenosides, gintonin)

The primary active molecules in ginseng are ginsenosides, also known as ginseng saponins. Ginseng has been shown to modulate pain through its effects on ion channels, specifically by influencing high-voltage-activated Ca^2+^ channels in rat DRG neurons. Total saponins from ginseng dose-dependently suppressed Ca^2+^ channel currents, particularly affecting L-, N- channels. Among ginseng's active components, ginsenoside Rg3 was identified as a key inhibitor of Ca^2+^ channels, likely contributing to ginseng's antinociceptive effects (73). Gintonin, extracted from ginseng root, modulates pain-related ion channels by inhibiting K_v_1.2 channel activity in a calcium-dependent manner. This inhibition, which involves phospholipase C and receptor protein tyrosine phosphatase α (RPTPα) pathways, highlights gintonin's potential role in regulating neuronal activity and pain signaling (74).

#### 3.1.13 *Glycyrrhiza uralensis* Fisch (licorice)

Licorice, made from roots of *Glycyrrhiza uralensis* Fisch, is used for its stomach and spleen-protective, pain-relieving, cough-alleviating, and phlegm-reducing effects (123). Licorice contains licochalcone A and licochalcone B, key compounds with potential analgesic effects. In DRG neurons, licochalcone A was found to inhibit Na_v_1.7 channel, reducing neuronal excitability, whereas licochalcone B, did not affect Na_v_. In animal models of formalin-induced pain, licochalcone A inhibited pain responses in both phases of the test, while licochalcone B, only reduced pain in phase 2. Licochalcones, particularly licochalcone A, could be promising candidates for developing Na_v_ channel-targeted analgesic drugs (75).

#### 3.1.14 *Ligusticum chuanxiong* Hort (ligustrazine)

*Ligusticum chuanxiong* Hort has long been used to treat cardiovascular conditions and related pain, including headaches, chest pain, and neuropathic pain (124). Ligustrazine, a primary active compound from *Ligusticum chuanxiong*, has demonstrated analgesic effects across various pain types, including angina, neuropathic, inflammatory, and burn pain, and has been shown to alleviate pain hypersensitivity caused by chronic venous disease in mice. Ligustrazine reduced pain responses to mechanical, cold, and thermal stimuli and desensitized TRPA1 channel activity in DRG neurons, thereby decreasing neuronal excitability (76).

#### 3.1.15 *Scutellaria baicalensis* (baicalein, wogonin)

The root of *Scutellaria baicalensis* has been widely used in Asia and the West for its health benefits, traditionally treating cardiovascular diseases, inflammation, and tumors. *Scutellaria baicalensis* is renowned for its high flavonoid content, containing four primary flavones: baicalin, baicalein, wogonoside and wogonin (125, 126). Baicalein and wogonin were found to activate the TREK-2 potassium channel, potentially contributing to neuroprotection. In COS-7 cells expressing TREK-2, both compounds increased channel opening frequency without affecting conductance or open time. Baicalein provided continuous activation, while wogonin activated TREK-2 transiently. These findings suggest that baicalein and wogonin may help regulate resting membrane potential (RMP) under pathological conditions, supporting their neuroprotective effects (77).

#### 3.1.16 *Sinomenium acutum* (sinomenine)

*Sinomenium acutum,* traditionally used for rheumatic arthritis and neuralgia, contains sinomenine, an active compound with immunosuppressive, anti-inflammatory, and analgesic properties that effectively alleviates both neuropathic and inflammatory pain (127, 128). Sinomenine, the active ingredient in *Sinomenium acutum*, shows analgesic effects in a formalin-induced inflammatory pain model in mice. Intraperitoneal administration of sinomenine reduced pain behaviors and suppressed c-Fos expression in the spinal cord. In DRG neurons, sinomenine increased the spike threshold, reduced firing frequency, and inhibited Na_v_ currents dose-dependently, suggesting that its peripheral analgesic effect involves inhibition of Na^+^ channels (78).

#### 3.1.17 *Sophorae radix* (sophora flavanone G)

*Sophorae radix*, derived from the roots of Sophora species, is traditionally used for pain relief and as an anti-inflammatory agent in conditions like arthritis (129). Ca_v_3.2 T-type Ca^2+^ channels are known for their role in pain signaling. Sophora flavanone G from *Sophorae Radix* and hop-derived analogues, (2S)-6-PNG and (2S)-8-PNG, are effective T-channel blockers. (2R/S)-6-PNG showed significant effects in reducing mechanical and visceral pain, as well as neuropathic allodynia in mice, without noticeable side effects on motor or cardiovascular function (79).

#### 3.1.18 *Pueraria montana* (puerarin)

Puerarin has shown analgesic effects in neuropathic pain models. It alleviates paclitaxel-induced pain by blocking Na_v_ channels, especially TTX-R Na_v_1.8 channels, in a β1 subunit-dependent manner. In CCI model, puerarin also reduced pain by downregulating P2X_3_ receptor expression in DRG neurons, increasing pain thresholds. These findings highlight puerarin's potential in managing neuropathic pain through modulation of Na_v_1.8 and P2X_3_ channels in sensory neurons (80, 81).

#### 3.1.19 *Paeonia lactiflora* (paenoiflorin)

*Paeoniflorin*, derived from the roots of *Paeonia lactiflora*, is widely used for pain relief and to treat conditions such as gynecological disorders, liver disease, neuroinflammation and rheumatoid arthritis (130, 131). Its anti-inflammatory, immunoregulatory, and antioxidant properties enhance its role in managing autoimmune diseases (132). *Paeoniflorin* acts as an analgesic by modulating ion channels involved in pain transmission, particularly through its stable binding to TRPV1, which directly suppresses the response of DRG neurons to capsaicin (82). It also inhibits L-type voltage-dependent calcium currents in a concentration-dependent manner and shifts the inactivation curve to more negative potentials in NG108-15 cells, indicating its role in modulating neuronal excitability and neuroendocrine functions (83). These channels regulate neuronal excitability and Ca^2+^ influx, which are crucial for nociceptive signal propagation. By inhibiting these channels, paeoniflorin effectively reduces pain perception and mitigates hyperalgesia, making it a potent agent for managing chronic and neuropathic pain.

#### 3.1.20 *Rhododendron molle* (grayanoids)

Grayanoids, derived from the dried roots of *Rhododendron molle*, are traditionally used for pain relief and modulate voltage-gated Na^+^ channels. They exhibit strong anti-nociceptive effects in pain models, such as the acetic acid-induced writhing, hot-plate, and formalin tests, with voltage-gated Na^+^ channels serving as key targets for their analgesic and toxic effects (133).

### 3.2 Stems&leaves

#### 3.2.1 *Artemisia annua* (artemisinin)

*Artemisia annua*, part of the Asteraceae family, has long been used in traditional medicine for malaria and fever treatment (134). Artemisia species and artemisinin exhibit various pharmacological effects, including antibacterial, antifungal, antioxidant and anti-inflammatory properties (135, 136). Artemisinin and its derivatives have shown significant pain-relieving effects by modulating ion channels like the P2X_4_ receptor in the DRG, associated with neuropathic pain. It demonstrated stronger antinociceptive effects, while artemisinin exhibited notable anti-inflammatory properties, reducing key pro-inflammatory cytokines (84, 137).

#### 3.2.2 *Boswellia carterii* and *Commiphora myrrha* (frankincense and myrrh)

Frankincense and myrrh are traditional resins used to relieve pain. Frankincense, from *Boswellia carterii*, may help regulate immune function, while myrrh, from *Commiphora myrrha*, has anti-inflammatory and antimicrobial effects (138, 139). Frankincense and myrrh, traditionally used together for synergistic pain relief, were studied for their mechanisms in neuropathic pain using mouse models. In a CCI model, a water extract of frankincense and myrrh effectively alleviated thermal hypersensitivity and mechanical allodynia (85). The studies highlighted the role of the TRPV1 receptor and the TLR4/MyD88 pathway in neuropathic pain. A water extract of frankincense and myrrh treatment reduced TRPV1 expression at both mRNA and protein levels and decreased calcium response in DRG neurons, while also inhibiting neuroinflammatory TLR4/MyD88 signaling in the spinal cord. These findings suggest that a water extract of frankincense and myrrh alleviates neuropathic pain by modulating TRPV1 and reducing neuroinflammation through the TLR4/MyD88 pathway, offering a potential approach for neuropathic pain treatment targeting ion channels and inflammatory signaling (86).

#### 3.2.3 *Camellia sinensis* (epigallocatechin gallate)

The leaves of *Camellia sinensis*, commonly consumed as green and black tea, contain polyphenols like epigallocatechin-3-gallate, a primary compound in green tea that has shown promise in preclinical studies for neuropathic pain treatment due to its anti-inflammatory and antioxidant effects, and has also demonstrated the ability to reduce bone cancer pain (140). In neuropathic pain caused by peripheral nerve injury, epigallocatechin-3-gallate and its derivatives were tested for analgesic effects. They effectively reduced thermal hyperalgesia long-term by inhibiting fatty acid synthase and lowering inflammatory protein levels, making it a promising candidate for neuropathic pain treatment in preclinical development (141). Epigallocatechin gallate inhibited ASIC3 currents effectively at low concentrations and reduced acid-induced pain behaviors in mice, highlighting its potential as a structural basis for developing pain-targeted drugs that modulate ASIC3 channels (87).

#### 3.2.4 Citrus reticulata

Citrus plants that have compounds including narirutin, naringenin, limonene, diosmetin, and newly studied TRPM3 blockers like Isosakuranetin, show promising potential for neuropathic pain management by targeting pain-related ion channels. Narirutin and Naringenin inhibit Na_v_1.7 and Na_v_1.8 Na^+^ channels, respectively, to reduce pain signaling. Limonene modulates TRPA1 channels, inducing pain topically but inhibiting pain systemically. Diosmetin acts as a TRPV1 antagonist, effectively reducing heat- and capsaicin-induced pain. Isosakuranetin, identified as a potent TRPM3 blocker, and hesperetin decrease responses to noxious heat and chemical pain in mice. Together, these compounds offer novel mechanisms for pain relief, targeting TRPV1, TRPA1, Na_v_1.7, Na_v_1.8, and TRPM3 channels, and highlight the potential for citrus-derived compounds in developing selective and effective analgesics (88, 89, 142–144).

#### 3.2.5 *Ephedra sinica* (ephedrine)

Ephedrine, derived from *Ephedra sinica*, *E. intermedia*, or *E. equisetina*, is known for its anti-inflammatory properties which contribute to its analgesic effects (145–147). These medicines are commonly used to treat pain conditions, primarily due to their anti-inflammatory actions. Interestingly, ephedra herb extracts not only activate the TRPV1 channel, a key mediator of pain sensation, but also induce its desensitization. This desensitization, observed *in vivo*, reduces capsaicin-induced pain by suppressing TRPV1 activity in peripheral sensory neurons, highlighting a dual mechanism where initial activation of TRPV1 is followed by a loss of channel sensitivity, ultimately leading to analgesia. These findings suggest ephedra herb extracts that may exert its pain-relieving effects through a combination of anti-inflammatory properties and modulation of TRPV1 signaling (90).

#### 3.2.6 Hericium erinaceus

The extracts and chemical components of the genus *Hericium*, a group of medicinal mushrooms traditionally used in herbal medicine, is actively progressing, revealing various pharmacological activities such as anticancer, antioxidant, anti-inflammatory, and nerve growth-promoting properties. Notably, *H. erinaceus* has gained attention for its potential to treat Alzheimer's disease, cancer, inflammation, depression, and nerve injury, highlighting its diverse health-promoting effects (148, 149). Erinacine-S, a small active component derived from *H. erinaceus* inhibits P2R-mediated Ca^2+^ signaling and reduces neuropathic pain and neuroinflammation in cell and mouse models through the modulation of P2X_4_ and P2X_7_. Ethanol extracts and erinacine-S showed potential for treating neuropathic pain (91).

#### 3.2.7 *Magnolia officinalis* (magnolol, honokiol, magnolin)

Magnolol, a polyphenolic compound from the bark of *Magnolia officinalis*, exhibits multiple pharmacological effects. It has anti-inflammatory properties by inhibiting NF-*κ*B, antioxidative effects useful for skin disorders, and anticancer effects in thyroid, bladder, and glioblastoma cells (150–152). Magnolol inhibited Na_v_ and K_v_ channels. These inhibitory effects on Na_v_ and K_v_ channels may contribute to magnolol's neuroprotective properties (92). Honokiol and magnolol, two active compounds from the bark of *Magnolia officinalis*, were tested for pain relief in mice. While they did not reduce pain in the tail-flick, hot-plate, or neurogenic phase of the formalin test, both compounds significantly reduced pain in the inflammatory phase of the formalin-induced response. They decreased formalin-induced c-Fos expression in the spinal cord's dorsal horn without affecting motor coordination or memory. These findings suggest that honokiol and magnolol may effectively treat inflammatory pain without causing motor or cognitive side effects (93). Magnolin, the major tetrahydrofurofuranoid lignan from *Magnolia denudata*, significantly alleviated paclitaxel-induced CIPN, which is characterized by sensory disturbances and neuropathic pain, through the suppression of ERK phosphorylation in the DRG (153).

#### 3.2.8 *Mentha arvensis* (menthol)

The leaves of *Mentha arvensis* are rich in menthol. Menthol, known for activating the TRPM8 channel to produce a cooling sensation, is widely used in topical analgesics (154). A previous study tested whether menthol also blocks Na_v_, which are critical in pain sensation. Results showed that menthol inhibits Na_v_1.8, Na_v_1.9, and TTX-S channels in a concentration-, voltage-, and frequency-dependent manner, promoting inactivation and reducing high-frequency neuronal firing. Low concentrations of menthol provided pain relief in mice, suggesting that its analgesic effect involves selective Na^+^ channel blockade (94). However, high doses of menthol increase neuron excitability by inhibiting leak K^+^ channels, likely K2P channels, in dural afferent neurons. This inhibition leads to membrane depolarization and lowers the threshold for AP generation, which may explain menthol's pronociceptive effect at high concentrations (155). That means, menthol's dual role in pain modulation, with lower doses providing analgesia and higher doses enhancing pain responses.

### 3.3 Fruits&flowers

#### 3.3.1 *Crataegus pinnatifida* (vitexin)

Vitexin, a C-glycosylated flavone (5, 7, 4-trihydroxyflavone-8-glucoside), is a primary bioactive compound in the traditional herb *Crataegus pinnatifida* (156). Vitexin reduces mechanical and thermal hyperalgesia and inhibits pain-like behaviors in various inflammatory pain models in mice. Its antinociceptive effects against inflammatory pain may be partially mediated by targeting the TRPV1 channel, reducing oxidative stress, and modulating cytokine production (95).

#### 3.3.2 Lycium barbarum

*Lycium barbarum*, commonly known as goji berry or wolfberry, has been widely used in medicine and contains bioactive compounds like polysaccharides, carotenoids, and betaine (157). The therapeutic effects of *Lycium barbarum* polysaccharides and capsaicin were investigated in a dextran sulfate sodium (DSS)-induced colitis model in rats. The treatments, administered via gavage, significantly reduced oxidative stress, inflammatory responses, and pain signaling. Specifically, both *Lycium barbarum* polysaccharides and capsaicin downregulated the expression of TRPV1 and TRPA1 ion channels in the colon, which are closely associated with pain and inflammation (96).

#### 3.3.3 *Inula britannica* (patuletin)

*Inula britannica*, traditional medicine for arthritis and back pain, was studied for its pain-relieving effects. The flower essential oil and its major component, patuletin, demonstrated significant antinociceptive effects in male mice across various pain models (tail-flick, writhing, formalin-induced, and glutamate-induced tests). Essential oil effects were reduced by opioid antagonists and blocked by methylene blue and glibenclamide, suggesting the involvement of opioid receptors and activation of the NO-cyclic GMP-protein kinase G/ATP-sensitive potassium channel signaling pathway (12, 122).

#### 3.3.4 *Garcinia mangostana* (α-mangostin)

*Garcinia mangostana* (mangosteen) has fruit-derived products used in traditional medicine for treating infections and reducing fever (158, 159). α-Mangostin, a primary xanthone from mangosteen pericarps, exhibits antioxidant, anti-inflammatory, and analgesic effects, likely by modulating ion channels in nociceptive neurons. α-Mangostin enhances K^+^ conductance, activates TREK/TRAAK channels, inhibits TRPV1 currents, and partially suppresses TTX-S Na_v_ channels, which reduces neuronal excitability and pain. This demonstrates that α-Mangostin exerts multi-target analgesic effects by modulating pain-related ion channels expressed in DRG neurons. Also, molecular docking and *in silico* ADME analyses support its stable interactions with these channels and its potential as a safe, multi-target analgesic agent without crossing the blood-brain barrier (97).

#### 3.3.5 *Tetradium daniellii* (pellitorine)

Pellitorine, from the fruits of *Tetradium daniellii,* as the first TRPV1 antagonist derived from the Evodia species. Through bioactivity-guided extraction and isolation, pellitorine blocks capsaicin-induced Ca^2+^ uptake. While other isolated compounds (e.g., N-isobutyl-4,5-epoxy-2E-decadienamide) showed no TRPV1 activity, pellitorine emerged as a competitive inhibitor. This compound, structurally analogous to capsaicin, may help inhibit inflammation-related pain (98). It also alleviated cold allodynia in an oxaliplatin-induced model (160).

#### 3.3.6 *Rhododendron molle* G. Don (Rhodojaponin III)

The flowers and fruits from *Rhododendron molle* G. Don, a traditional medicinal herb, are well-known for their pain-relieving properties. Rhodojaponin III, the primary active and toxic component extracted from this plant, has been investigated for its antinociceptive effects, underlying mechanisms, and subacute toxicity. Rhodojaponin III showed significant pain-relieving effects in nociceptive pain models, including hot plate, tail-immersion, acetic acid writhing, and formalin tests, as well as reduction in hyperalgesia in a CCI model. Molecular docking and electrophysiological studies revealed that Rhodojaponin III mildly inhibits Na_v_1.7, Na_v_1.8, and Na_v_1.5 (99).

## 4 Discussion

Pain management remains a complex and persistent challenge in both clinical and research settings, primarily due to the multifaceted nature of pain signaling pathways and the diverse origins of pain conditions. Opioids, NSAIDs, and gabapentin are commonly used pharmacological therapies, widely recognized for their effectiveness in pain relief. However, their uses often come with significant side effects. Opioids are associated with risks of addiction and tolerance (3, 6); NSAIDs can lead to gastrointestinal irritation, cardiovascular events, and renal dysfunction (7, 9); Gabapentin, while effective for neuropathic pain, may cause dizziness, sedation, and dependency in some cases (5, 10, 11). These adverse effects not only limit the long-term usability of these medications but also highlight the necessary need for alternative therapeutic strategies. These challenges underscore the importance of exploring alternative therapeutic strategy that can effectively manage pain while offering improved safety profiles.

**Figure 1:**
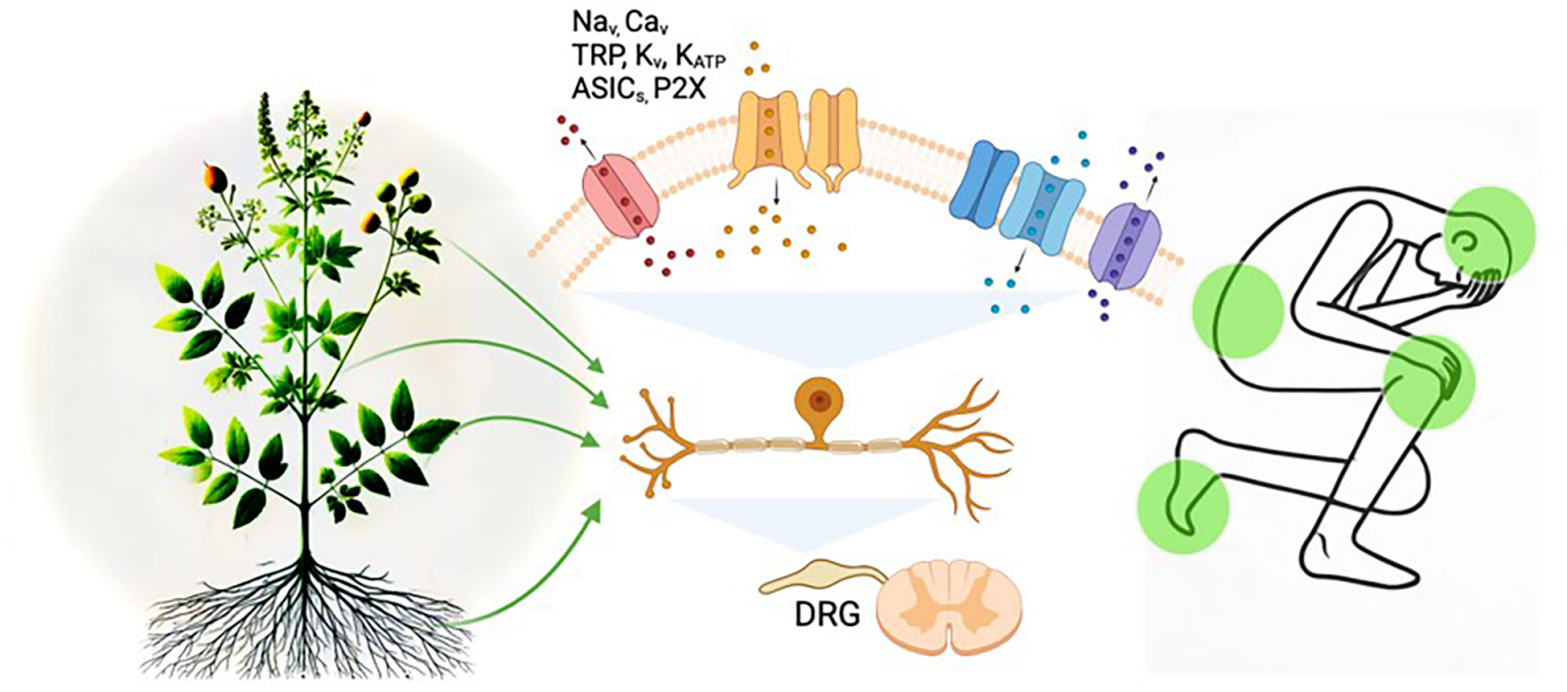
Diagram of mechanisms linking plant-derived medicines, ion channels, and pain modulation. Illustrating the potential mechanisms of pain modulation by plant-derived compounds through sensory neuronal ion channels. The image depicts how bioactive compounds from plants may target ion channels, such as Na^+^, Ca^2+^, TRP, K_v_, K_ATP_ ASICs, and P2X, in dorsal root ganglion (DRG) neurons to alleviate pain signals. The integration of natural products and ion channel regulation in pain pathways is highlighted.

Medicinal herbs may offer a promising solution to this issue. These herbs have been performed for their analgesic properties in traditional medicine, and recent advancements in scientific research have begun to unravel their molecular mechanisms of action (Figure 1). Plant-based compounds derived from a single herb often include multiple extracts, each contributing to analgesic effects. Even a single phytochemical adopts a multi-targeted approach, rather than targeting a single ion channel as seen in conventional single-target therapies (68, 82, 83, 89, 97). For instance, α-mangostin applied to DRG neurons significantly inhibited TRPV1 currents in the micromolar (µM) range and TTX-S Na_v_ currents in the millimolar (mM) range. Furthermore, α-mangostin activated K2P channels in the µM range, hyperpolarizing the RMP of DRG neurons (97). This highlights that a single molecule like α-mangostin can exert broad effects by simultaneously modulating various ion channels associated with pain transmission, providing comprehensive and potent pain relief. Since pain involves complex pathophysiological mechanisms, targeting a single pathway is insufficient to address and treat it fully. From this perspective, the diverse analgesic mechanisms of natural compounds make them a promising option for treating multifaceted pain conditions.

Interestingly, different parts of a plant, such as fruits, flowers, stems, leaves, and roots, are rich in varying levels of phytochemicals with analgesic properties, making them promising candidates for pain management applications. Fruits and flowers, include flavonoids, phenolic acids, essential oils, and alkaloids (161, 162). Stems and leaves commonly harbor tannins and terpenoids (163, 164), and roots frequently contain saponins and polysaccharides (67, 165). Understanding these specific phytochemical profiles allows for more targeted research and application in pain management. Moving forward, the botanical and pharmaceutical industries are likely to emphasize the systematic utilization of these plant components, optimizing extraction and formulation methods to maximize their therapeutic potential in analgesics.

Despite their potential, only a small fraction of herb-related compounds has successfully been translated into clinical practice. Among these, certain herbal medicines have demonstrated clinical efficacy as analgesics. For example, curcumin, widely used for managing pain and inflammation, is safely administered at 400–600 mg of standardized powder up to three times daily or 1–3 g of dried powdered root (166, 167). Similarly, clinical trials recommend 100–250 mg of *Boswellia carterii* extract daily for at least 4 weeks to improve pain, stiffness, and joint function (168). Menthol, commonly used to alleviate cold symptoms such as nasal congestion and nighttime cough, is safely applied topically in ointments (5–10 ml) to the chest and neck, making it suitable for individuals aged 2 years and older (169). However, several factors limit the broader clinical application of these compounds, with one significant challenge being the lack of standardization for active compounds. The multi-component and complex nature of herbal extracts make it difficult to standardize specific active ingredients and clearly define their effects (170–173).

Integrating medicinal herbs into recent clinical pain management protocols remains a formidable challenge. Key areas requiring further research include the standardization of dosages, ensuring the consistency and quality of herbal extracts, and optimizing delivery methods to maximize therapeutic benefits. Moreover, large-scale clinical trials are essential to validate the efficacy and safety of these herbal compounds across diverse patient populations. Addressing these issues could pave the way for the successful clinical translation of medicinal herbs, providing safer and more effective alternatives for pain management.
